# Looking up to virtue: averting gaze facilitates moral construals via posteromedial activations

**DOI:** 10.1093/scan/nsy081

**Published:** 2018-09-13

**Authors:** Xiao-Fei Yang, Gabriela Pavarini, Simone Schnall, Mary Helen Immordino-Yang

**Affiliations:** 1 Brain and Creativity Institute, University of Southern California, Los Angeles, CA, USA; 2 Rossier School of Education, University of Southern California, Los Angeles, CA, USA; 3 Department of Psychiatry, University of Oxford, Oxford, OX1 2JD, UK; 4 Department of Psychology, University of Cambridge, Cambridge, CB2 3EB, UK; 5 Department of Psychology, Neuroscience Graduate Program, University of Southern California, Los Angeles, CA, USA

**Keywords:** admiration, construal level theory, default mode network, abstract cognition, moral elevation

## Abstract

Witnessing exemplary actions triggers admiration, a positive emotion that can pertain to concrete skills, or move the onlooker beyond physical characteristics to appreciate the abstract, moral implications. Participants reacted to narratives depicting skilled or virtuous protagonists first during a videotaped interview then during functional magnetic resonance imaging (fMRI). We analyzed participants’ gaze aversion (an indicator of disengaging from the immediate environment) and cognitive construals (mentions of concrete characteristics *vs* abstract beliefs and values) during the interview, and relations to individuals’ subsequent neural activations. When participants averted their gaze, they were more likely to mention abstract construals, and both behaviors were more likely when reacting to virtue. Gaze aversion to virtue narratives predicted greater subsequent activation for those narratives in dorsal posterior cingulate cortex (dPCC), involved in visual attention control. The inferior-posterior posteromedial cortices (ipPMC), a default mode network hub involved in abstract thought, activated only to virtue, and activity level reflected individuals’ tendency to abstract construals. Critically, dPCC and ipPMC activity sequentially mediated the relationship between individuals’ gaze and abstract construal tendencies. No such relationships existed for reactions to skill, despite participants reporting equivalently strong positive emotion. In appreciating virtue, dPCC may support individuals in transcending the viewable context, facilitating ipPMC activity and moral construals.

## Introduction

Witnessing other people’s exemplary actions triggers strong positive feelings that inspire us to better ourselves (Haidt, 2003). However, people’s outstanding actions encompass many different domains, ranging from expressions of bravery, compassion or self-sacrifice to demonstrations of high competence and skill. Is the moral virtuoso evaluated in the same way as the physically or cognitively skilled virtuoso?

A major focus of social neuroscience research has been on the role of empathic action simulation in understanding and learning from others (Gallese *et al*.,  2004; Iacoboni,  2009). However, while admiring others’ skills involves attention to the physical context, appreciating someone else’s exemplary moral actions involves moving beyond the observable details of their actions to engage in abstract inferences about the target’s psychological qualities and the broader social implications (Bruner, 1986). For example, the actions of a civil rights leader are admirable not for their physical attributes but for what they may reveal about his or her moral convictions, bravery and dedication to an important cause. Such cognitive abstractions in turn lead to reflections about general values and beliefs that can be profoundly motivational (Dewey, 1933; Immordino-Yang and Sylvan, 2010), and can provide the impetus for engaging in costly action to the benefit of others in need (Haidt, 2003; Schnall *et al*., 2010; Schnall and Roper, 2012). Individuals vary in the degree to which they engage abstract interpretations, and much effort is devoted in secondary education to cultivating this beneficial habit of mind (Nickerson *et al*., 1985; Baehr, 2013; Ritchhart, 2015).

At the behavioral level, one basic means through which individuals facilitate abstract cognition is by transcending the immediate environment through looking away. Individuals gaze away from their interlocutor and from other types of visual displays when concentrating on difficult cognitive tasks that go beyond the physical here-and-now, such as trying to remember information (Doherty-Sneddon *et al*., 2002; Doherty-Sneddon and Phelps, 2005). Doing so improves their performance (Glenberg *et al*., 1998; Phelps *et al*., 2006; Markson and Paterson, 2009). In social contexts, gaze aversion may be particularly instrumental to abstract cognition, as direct eye contact can be affectively arousing (Helminen *et al*., 2011) or even threatening (Schulze*et al*., 2013).

Given this research, one would expect individuals to spontaneously look away while making meaning of others’ moral actions. Although, to our knowledge, no available research has tested this hypothesis, recent findings suggest connections between eye movements and both moral decision-making (Pärnamets *et al*., 2015) and introspection (Reichle *et al*., 2010; Smilek *et al*., 2010). In the same vein, a recent study suggested that moral leaders (e.g. Martin Luther King, Jr) are often portrayed as gazing away (up and right; Frimer and Sinclair, 2016).

At the neural level, engagement with events beyond those that occur in the immediate environment has been consistently linked with heightened activity in brain regions including the posteromedial cortices (PMCs; Raichle *et al*., 2001; Buckner and Carroll, 2007; Spreng *et al*., 2009), an ensemble that includes the posterior cingulate, precuneus and retrosplenial cortices. The PMC has extensive long-range connections with other association cortices (Parvizi *et al*., 2006; Hagmann *et al*., 2008; Cauda *et al*., 2010) and is considered the most centrally connected structural core of the brain (Hagmann *et al*., 2008), making it ideally positioned to integrate information across the cerebral cortex to support complex mental abstraction (Damasio, 1989; Meyer and Damasio, 2009). Indeed, the inferior-posterior sector of the PMC (ipPMC) is a key hub of the default mode network (DMN; Andrews-Hanna *et al*., 2010), a group of distributed but functionally interconnected brain regions posited to support abstract internally focused thoughts (Immordino-Yang *et al*., 2012), such as thinking about values and beliefs (Kaplan *et al*., 2016a,b) and judging the morality of others’ actions (Greene *et al*., 2001; Reniers *et al*., 2012). Of particular relevance to our investigation, reacting to others’ expressions of virtue was found to preferentially activate the ipPMC (Immordino-Yang *et al*., 2009). The ipPMC was not especially recruited in reaction to acts that are skillful but not morally meaningful (e.g. skillful painting or sports playing), which instead preferentially activated the superior–anterior sector of the PMC (Immordino-Yang *et al*., 2009). Indeed, the superior–anterior PMC is closely linked with sensorimotor and musculoskeletal processing (Parvizi *et al*., 2006; Cauda *et al*., 2010) and is not a part of the DMN (Margulies *et al*., 2009).

Interestingly, a dorsal region of the posterior cingulate cortex (dPCC), adjacent to the ipPMC, is known to be involved in visual attentional control (Dean *et al*., 2004; Cauda *et al*., 2010). While the dPCC is strongly connected with the DMN, it is also part of the frontal-parietal control network (Leech *et al*., 2011) involved in executive attentional control (Vincent *et al*., 2008). Given its unique connectivity, the dPCC has been proposed to play an important role in directing attention focus internally or externally in reaction to environmental stimuli by tuning whole brain network stability (Leech and Sharp, 2014).

### Goals and hypotheses

The neural organization, together with evidence that gaze aversion facilitates abstract cognition, suggests individuals may avert their gaze from immediate social visual information to facilitate the abstract, introspective cognitive processing that is relevant to appreciating the moral implications of exemplary actions. Although the past decades have seen a remarkable effort to elucidate the neuropsychology of abstract thinking and morality (Greene *et al*., 2001; Young and Saxe, 2008; Kaplan*et al*., 2016a), to our knowledge the proximal mechanism through which individuals adopt an introspective, abstract mindset has not yet been documented. Here we investigated participants’ verbalized cognitive construals, gaze aversion and PMC neural activation as they reacted to true narratives depicting virtuous *vs* skilled protagonists. In both conditions, participants engaged with another person’s exemplary actions; however, whereas one can directly observe skills, appreciating a protagonist’s virtue—their moral beauty of character—involves drawing conclusions in light of abstract norms, values and beliefs that are not directly observable.

Participants were exposed to compelling videos based on the lives of real people, first during a videotaped interview (to measure cognitive construals and eye gaze) and then during blood oxygen-level dependent functional magnetic resonance imaging (BOLD fMRI). Target narratives were about virtuous people (e.g. an ex-gang member who reforms and opens a prison-to-work training program) and skilled people (e.g. a young man skillfully breakdancing). The interview procedure resembles real-life storytelling interactions, which are ubiquitous across human cultures and a primary way through which achievements, beliefs and values are transmitted (Tappan and Brown, 1989; Merrill and Fivush, 2016).

We first tested whether reacting to virtue narratives selectively activated the ipPMC, whereas reacting to skill narratives selectively activated the superior–anterior PMC sector, replicating Immordino-Yang *et al*., (2009). We then examined whether, during the interview, (1) participants were more likely to avert their gaze when reacting to virtue than when reacting to skill and whether (2) reactions to virtue involved more abstract construals (i.e. references to values and beliefs) and fewer concrete construals (i.e. references to physical/cognitive abilities and traits) than did reactions to skill. Next, we analyzed whether, for narratives to which a participant had averted their gaze, (3)(a) they were more likely to produce an abstract construal, and (b) they showed higher dPCC activity when reacting to that stimulus during fMRI. Finally, we tested whether in the virtue condition, (4)(a) individuals who produced more abstract construals also averted their gaze more overall, and, if so, whether (b) dPCC and ipPMC activity levels mediated this tendency.

## Methods

Data for the current study were collected as part of a larger project investigating lifespan development of social emotion processing and its neurobiological correlates in multiple cultural contexts (*n* = 143). From the larger project sample we selected all young adults (aged 18–30) who had been born and raised in the USA. We restricted our sample to Americans to avoid complications due to translation in the coding of cognitive construals. We restricted to adults because adolescents are not yet reliable in their ability to think abstractly (Fischer and Bidell, 2006). We did not include adults over the age of 30 to avoid confounds from possible age-related changes in attention (e.g. Verhaeghen and Cerella, 2002). We excluded six otherwise qualified participants whose data had been collected for Immordino-Yang *et al.*(2009) as we sought in part to replicate that analysis. The sample partially overlaps with the sample used in Immordino-Yang *et al*. (2014, 2016), which describe unrelated aspects of the data set.

### Participants

The sample included 32 right-handed volunteers (16 females; average age 21.1 years, *SD* = 2.60) who participated and were compensated in accordance with institutional review board requirements. A total of 16 participants self-identified as Asian-American, 12 as Caucasian-American, 2 as Latino-American and 2 as African-American. All participants spoke English as their first language. There were no data exclusions.

### Procedure

The study utilized a previously developed, systematic and controlled procedure (Immordino-Yang *et al*., 2009) that allowed us to observe individual variability in naturally emerging verbal and non-verbal behavior while maintaining a high level of experimental control. Participants were individually interviewed by the same female experimenter about their reactions to 20 true stories (10 for each content type) depicting virtuous and skilled others. Participants were also exposed to 30 additional stories meant to induce varieties of compassion/empathy and unemotional social processing; because they are not relevant to the current investigation of construals of exemplary actions these data are not included. Treating these stimuli as filler items had the benefits of disguising the specific goal of the current investigation and preventing habituation to the admiration stimuli.

The experimenter presented each narrative orally using a memorized script that began, ‘This is a story of a man/woman who…’, and included a brief description of the protagonist’s circumstances and accomplishments, followed by supplementary video images of the narrative protagonist shown on a laptop computer. Virtue narratives included, for example, the story of a woman who adopted 10 special-needs children. Skill narratives included, for example, a young woman who solved a Rubik’s Cube blindfolded after memorizing the cube’s starting configuration. These stories were matched for length and complexity and had been extensively pre-tested (for details, see Immordino-Yang *et al*., 2009; Supplementary Information). Narratives were presented in one of two counterbalanced fixed pseudo-random orders in a 2 h videotaped interview session. Participants were not told what reactions the narratives were intended to elicit.

Each narrative took between 60 and 90 s to present, and at the end of each, the video image froze and stayed a static still image for the remainder of the trial (i.e. while the participant described his/her reaction). After the video ended, the experimenter asked, ‘How does this person’s story make you feel?’ The experimenter then unobtrusively gazed downward into her notebook and transcribed some words from the participant’s response, which was explained as taking notes in case the video camera failed. In reality this note-taking was intended to systematize the experimenter’s gaze direction so that she would not inadvertently influence the behavior of the participant during the response phase. This scripted paradigm also ensured that participants’ spontaneous gaze aversion would not reflect avoidance of eye contact or, alternately, seeking of social approval from the experimenter.

Immediately after the interview, participants underwent BOLD fMRI as they viewed 5 s videos depicting the crux of each narrative with one sentence in both auditory and written forms. Each video was followed by 13 s of gray screen. They were instructed to think about the complete narrative that they had been told earlier and to become as emotional as possible. For each trial, participants reported via button press how strong of an emotional reaction they felt, which ensured compliance with the instructions. A fixation cross appeared for 2 s to separate trials. Each narrative was shown twice over the course of the fMRI experiment, but never twice during the same run, for a total of 100 trials divided into 4 runs of approximately 9 min each.

### Coding of manipulation check and cognitive construals

The videotaped interviews were later transcribed by raters blind to the study hypotheses, and transcriptions were independently verified by a second rater. Based on the transcripts, participants’ verbal responses to each story were coded for the presence (score of 1) or absence (score of 0) of positive affect, abstract construals and concrete construals. We adopted a dichotomous coding scheme to control for utterance length. (Information about gaze was not available to transcript coders.) Each answer was coded for the following:

- Positive affect (Manipulation Check): Descriptions of positive emotional feelings and praise for the protagonist. This category was included to establish that both types of stimuli were experienced as highly positive, to therefore control for the possible confound of affect. Examples included: ‘Amazed’, ‘inspired and happy’ and ‘incredibly impressed’.
- Values and beliefs (Abstract Construals): Descriptions of general beliefs, values and attitudes. Examples included: ‘It’s always good to give back to the community. Just learn from your mistakes, and just to do better’; ‘I think it just proves that people can definitely rise above their situations no matter how bad it is’; ‘she gives me hope for humanity’; ‘more people should be as dedicated to helping others’.
- Physical skills and characteristics (Concrete Construals): Statements describing physical characteristics or suggesting contrasts between characteristics of the participant and those of the protagonist in the form of statements about the self, envy, jealousy or the participant wishing he or she possessed the protagonist’s ability. Examples included: ‘I can’t dance. I have the skills and muscular coordination of a sledgehammer’; ‘people with that kind of talent make me so jealous’; and ‘I kinda wish I could do that, be that [physically] flexible’.

### Coding of gaze aversion

During the interview the participant was seated to the right of the experimenter at a table, so that both could comfortably watch the video materials on the laptop directly in front of the participant. Because we were interested to study spontaneous gaze ‘away’ and had no a priori reason to think that gaze left *vs* right would have psychological significance, we chose to standardize the experimental setup rather than counterbalance rightward and leftward gaze aversion. To the right of the participant was a nondescript office wall; above was a white ceiling.

Participants’ eye gaze while describing their reaction to each narrative was coded from the video with the sound turned off. The coder was blind to stimulus content. By default, participants would start describing their feelings looking frontward and down to the computer screen depicting the narrative materials or toward the experimenter (seated at their left). Thus, participants received a score of 0 for each narrative response in which gaze remained either toward the experimenter or on the computer screen. Gaze aversion was defined as shifting gaze upward (toward the blank ceiling) and/or to the right (toward the nondescript wall) while responding, in which case participants received a score of 1 for that trial.

### Coding reliability

A sample of 20% of the transcripts and videos were independently coded by a second rater blind to conditions and hypotheses; inter-rater agreement was high: Cohen’s Kappa ranged from 0.73 to 1.00 for the various types of coded content. Thus, remaining analyses were performed using the original rater’s codes.

### MRI data acquisition and pre-processing

Neuroimaging data were collected at the University of Southern California Dana and David Dornsife Neuroimaging Center. Whole brain images were acquired using a Siemens Tesla MAGNETOM TIM Trio scanner (Siemens Medical Solutions USA, Inc.) with a 12-channel matrix head coil. Functional scans were acquired using a T2^*^-weighted echo-planar imaging (EPI) sequence (TR = 2 s, TE = 30 ms, flip angle = 90°, acquisition matrix: 64 × 64, FOV = 192 mm) with a voxel resolution of 3 × 3 × 4.5 mm. We utilized Prospective Acquisition Correction to automatically correct for motion during data acquisition. Thirty-two continuous transverse slices were acquired to cover the whole brain and brain stem. Anatomical images were acquired using a magnetization-prepared rapid acquisition gradient echo sequence (TI = 900 ms, TR = 1950 ms, TE = 2.26 ms, flip angle = 7°, isotropic voxel resolution of 1 mm; acquisition dimensions: 256 × 256 × 160).

Neuroimaging data were processed using SPM8 (Wellcome Department of Cognitive Neurology, London, UK) in MATLAB 2009b (MathWorks, Inc., Natick, MA, USA). Functional images were slice timing corrected, motion corrected and co-registered to the anatomical image. Co-registrations were individually examined for each participant in native space to ensure high-quality alignment. The anatomical images were normalized to Montreal Neurological Institute (MNI) space using the segmentation procedure. The resulting normalization transformation was applied to the functional images, which were then resampled into an isotropic voxel resolution of 2 mm and smoothed using an 8 mm full width at half maximum Gaussian kernel.

### Modeling neural responses to narrative stimuli

Following Immordino-Yang *et al*. (2009), for each participant, each narrative condition was modeled using a finite impulse response function with nine time bins (each bin corresponding to a 2 s TR) to capture the complex neural activity during the 18 s trial. To calculate emotion-related BOLD response, parameter estimates over the 4th–8th time bins (6–16 s post-trial onset) were averaged for the virtue and the skill condition separately.

**Fig. 1:**
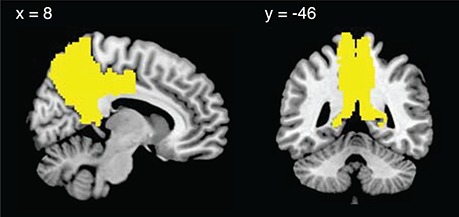
Sagittal and coronal views of the anatomically defined PMC mask.

Average BOLD responses to virtue stimuli and to skill stimuli were contrasted at the individual level and then tested at the group level for consistent effects. Results were examined within an anatomically defined PMC mask (Figure 1) and thresholded using the False Discovery Rate correction at *q*(FDR) < 0.01.

### Identifying the regions of interest

The regions of interest (ROIs) were functionally defined based on significant results from the virtue *vs* skill contrast describe above. The ipPMC ROI was defined as a 6-mm sphere centered at the peak of the ipPMC significant cluster. Because the dPCC significant cluster has two lateralized local maxima, the dPCC ROI was defined as the union of two 5-mm spheres, one centered at the left local maximum and the other centered at the right local maximum. The sphere radii were chosen to ensure that the ipPMC and dPCC ROIs were comparable in size.

Average neural activity from voxels within the ROIs was extracted using the MarsBar toolbox in SPM (Brett *et al*., 2002). Emotion-related BOLD responses were calculated using the same methods as described in ‘Modeling neural responses tonarrative stimuli’*.* For the trial-by-trial analyses, responses for the 2 presentations of the same narrative stimulus were averaged, resulting in 20 values per participant, 1 for each narrative stimulus. For the individual difference-level analyses, responses for narratives within each of the target conditions were averaged, resulting in two values per participant, one for virtue and one for skill.

### Modeling nested data using generalized estimating equation models

Because our data consisted of observations (i.e. responses to stories) nested within participants, unless otherwise specified, our analyses are based on generalized estimating equation (GEE) models (SPSS 18, SPSS Inc., Chicago, IL, USA). GEE models provide unbiased parameter estimates that account for within-subject correlation of repeated measure responses (Ballinger, 2004). Binary logistic response functions were used for binary behavioral outcomes (i.e. presence or absence of positive affect, gaze aversion and abstract and concrete construals). Linear scale response functions were used for analyses involving neural responses and individual difference-level measures.

### Testing mediation

Mediation of the individual differences was tested using the bias-corrected bootstrapping procedure described in (Preacher and Hayes, 2008; PROCESS v2.16) and implemented in SPSS 18 (SPSS Inc., Chicago, IL, USA).

## Results

### Manipulation check

To first confirm that the two types of narratives elicited strong positive affect, we compared self-reported positive emotions in the interview. Elements of praise and positive affect were observed in response to 98% of the virtue trials and 97% of the skill trials. The small difference between the conditions was not significant (*b* = 0.81*,SE* = 0.48, *p* = 0.09).

### Contrasting reactions to skill and virtue

Replicating our previous findings (Immordino-Yang *et al*., 2009), reacting to virtue elicited more activation in the ipPMC. Reacting to skill elicited more activation in the anterior/superior portion of the PMC and the dPCC (Table 1).

**Table 1:** Voxel clusters within the anatomically defined PMC mask whose BOLD activity differed significantly between the virtue and the skill conditions, in MNI space, thresholded at *q(FDR)* < 0.01

Brain region	Coordinates			*z*-score	cluster size
	*x*	*y*	*z*		
**Virtue > Skill**						
ipPMC	2	−62	28	6.41	700
**Skill > Virtue**						
Anterior/superior PMC	−14	−66	58	5.85	317
	14	−60	62	5.88	362
dPCC	−10	−34	42	4.56	496
	10	−36	46	3.73	Same cluster

Notes: dPCC: dorsal posterior cingulate cortex; ipPMC: inferior-posterior sector of the posteromedial cortices.

**Fig. 2:**
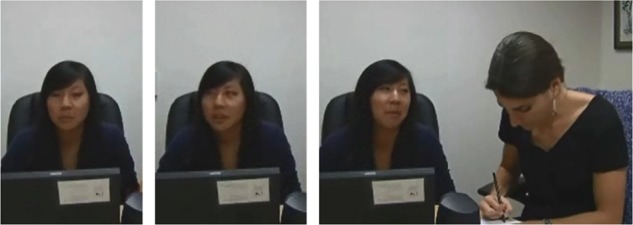
Still images from a 20 s segment of a participant’s interview, illustrating gaze aversion. The participant watches the video (first frame) then averts her gaze toward a nondescript wall on her right while thinking about her emotional reaction (second frame) then describes how she feels to the experimenter (third frame). The experimenter looks down at her notebook throughout.

We then confirmed that participants were more likely to avert their gaze (hypothesis 1; Figure 2) and were more likely to produce abstract construals and less likely to produce concrete construals (hypothesis 2) in virtue than in skill trials (Table 2).

**Table 2:** Frequencies (in percentage of trials) of gaze aversion and cognitive construals in response to the virtue and skill stimuli, and GEE model statistics comparing the two conditions

	Virtue	Skill	GEE statistics comparing virtue to skill			
			*b* (*SE*)	*Exp(b) *odds ratio	*95%* CI of odds ratio	*p*
Gaze aversion	54.7%	35.9%	0.77 (0.17)	2.15	[1.56, 2.98]	<0.001
Abstract construals	30.6%	14.7%	0.94 (0.16)	2.56	[1.88, 3.50]	<0.001
Concrete construals	5.9%	24.4%	−1.63 (0.24)	0.20	[0.12, 0.32]	<0.001

### Trial-by-trial relationships of gaze aversion to abstract construals and to dPCC activation

In the interview, participants were more likely to produce abstract construals in trials during which they had averted their gaze (hypothesis 3a; *b* = 0.80, *SE* = 0.28, odds ratio = 2.22, 95% CI of odds ratio [1.29, 3.81], *p* = 0.004). There was no interaction by stimulus condition (*b* = 0.16, *SE* = 0.16, *p* = 0.32).

Gaze aversion to a particular interview trial predicted subsequent dPCC activation to the same stimulus differently by condition (*b* = 0.06, *SE* = 0.03, 95% CI [0.001, 0.12], *p* = 0.047): gaze aversion to a virtue stimulus predicted higher dPCC activation to that stimulus (hypothesis 3b, *b* = 0.08, *SE* = 0.06); gaze aversion to a skill stimulus predicted lower dPCC activation (*b* = −0.04, *SE* = 0.04).

### Individual difference-level results

See Table 3 for descriptive statistics.

**Table 3:** Descriptive statistics of individual difference-level gaze aversion and cognitive construal scores for the virtue and the skill conditions

	Virtue			Skill		
	Mean (out of 10)	*SD*	Range	Mean (out of 10)	*SD*	Range
Gaze aversion	5.47	3.76	0–10	3.59	3.06	0–10
Abstract construals	3.06	2.02	0–6	1.47	1.52	0–5
Concrete construals	0.59	0.76	0–2	2.44	2.03	0–8

In the virtue condition, participants who showed more gaze aversion also produced more abstract construals (hypothesis 4a; *b* = 0.25, *SE* = 0.07, 95% CI [0.11, 0.40], *p* < 0.001). No such relationship was found for skill (*b* = 0.08, *SE* = 0.09, 95% CI [−0.10, 0.26], *p* = 0.36). There was a significant interaction between the conditions (*b* = 0.18, *SE* = 0.08, 95% CI [0.01, 0.34], *p* = 0.04). In contrast, there was no main effect of gaze aversion score on concrete construals, and no interaction by condition (*p* > 0.15).

In the virtue condition, participants who showed more gaze aversion showed higher dPCC activation (*b* = 0.11, *SE* = 0.04, 95% CI [0.24, 0.19], *p* = 0.01). No such relationship was found for skill (*b* = −0.04, *SE* = 0.04, 95% CI [−0.12, 0.04], *p* = 0.28). There was a significant interaction between the conditions (*b* = 0.15, *SE* = 0.05, 95% CI [0.05, 0.25], *p* = 0.003).

Participants who produced more abstract construals showed higher ipPMC activation (*b* = 0.22, *SE* = 0.08, 95% CI [0.05, 0.38], *p* = 0.01), with no interaction by condition (*b* = 0.004, *SE* = 0.11, 95% CI [−0.22, 0.23], *p* = 0.97).

**Fig. 3:**
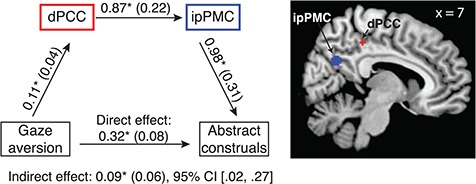
In the virtue condition, average activation in the dPCC and ipPMC sequentially mediated the relationship between individuals’ tendency to avert their gaze and to produce abstract construals. Regression coefficients for each path and the bootstrapped indirect effect are depicted with standard errors in parentheses. ^*^p < 0.05. dPCC and ipPMC ROIs are shown on the right. Notes: dPCC: dorsal posterior cingulate cortex; ipPMC: inferior-posterior sector of the posteromedial cortices.

For the virtue stimuli, the relationship between gaze aversion and abstract construals was mediated sequentially by dPCC activation and ipPMC activation (hypothesis 4b). The indirect effect determined using 5000 bootstrapped samples was 0.09 with standard error 0.06; the 95% confidence interval ranged from 0.02 to 0.27 (statistically significant because it does not cross zero; Figure 3). No such effect was found for the skill condition.

## Discussion

Our findings show that, though individuals experienced strong positive emotion to both virtuous and skilled protagonists, these reactions differed in both their psychological content and their neural underpinnings. More specifically, reactions to virtue tended to be more abstract, invoking general values, beliefs and moral ideals, while reactions to skill tended to be more concrete, invoking direct evaluations of physical characteristics and abilities, often in comparison to one’s own. Consistent with previous work (Immordino-Yang *et al*., 2009), reacting to virtuous protagonists preferentially activated the ipPMC, a key hub of the DMN (Andrews-Hanna *et al*., 2010) implicated in internally focused, value-oriented thinking (Greene *et al*., 2001; Immordino-Yang *et al*., 2012; Kaplan *et al*., 2016a,b), episodic memory and self-processing (Summerfield *et al*., 2009; Spreng and Grady, 2010; Araujo *et al*., 2014). Reacting to skilled protagonists preferentially activated the anterior/superior sector of the PMC, linked to sensorimotor and musculoskeletal processing (Parvizi *et al*., 2006; Cauda *et al*., 2010).

Our results also suggest that abstract construals of others’ actions are facilitated by averting one’s gaze. While the admirable features of a skilled performance can be directly observed, the admirable features of a virtuous performance cannot and must be constructed internally by the onlooker. We found that exposure to actions with virtuous implications was more likely to elicit gaze aversion, a non-verbal indicator of distancing from the immediate surroundings, and that regardless of whether a particular narrative depicted virtue or skill, averting one’s gaze when reacting to it increased the likelihood of an abstract construal.

The dPCC may play a role in this facilitation process. The dPCC, involved in visual attention control (Dean *et al*., 2004; Cauda *et al*., 2010; Leech and Sharp, 2014), is a shared node between the frontal-parietal control network and the DMN (Leech *et al*., 2011). When individuals shift their gaze away from a virtuous protagonist, the dPCC may contribute to stabilizing DMN activation, thereby offloading social visual information processing and facilitating a shift to abstract thought. In our study, gaze aversion to a particular virtue stimulus predicted increased dPCC activation to that stimulus. Individuals with stronger tendencies to avert their gaze when reacting to virtue showed a tendency toward abstract construals of virtue narratives, a relationship that was sequentially mediated by dPCC and ipPMC activation levels.

Notably, the dPCC was also active during reactions to skill, and in fact more so than during reactions to virtue. However, the relationship between gaze aversion and dPCC activity differed by condition. Unlike for virtue, averting one’s gaze to a skill stimulus predicted lower subsequent dPCC activation. In addition, at the individual-difference level, dPCC activity level in the skill condition did not play a role in mediating the relationship between gaze aversion and abstract construals. Consistent with its dual role in the frontal-parietal control network and the DMN, dPCC activation in the skill condition possibly reflects outwardly focused attention by shifting gaze ‘toward’ the target’s actions*,* which are enthralling to watch. Indeed, other researchers have suggested that admiration for skill should be linked to prolonged stares to facilitate learning (Henrich and Gil-White, 2001; Onu *et al*., 2016). Future studies should investigate the role of dPCC and gaze in other types of effortful internally focused mental processes, such as struggling with conceptual understanding of complex information (Nickerson *et al*., 1985). If dPCC is similarly implicated, the present results would represent an example of a more general mechanism through which dPCC manages visual attention to facilitate concrete and abstract modes of thought.

These results speak to a longstanding philosophical debate about the nature of moral expertise, and whether moral and physical/cognitive excellence is evaluated differently (Snow, 2015; Stanley and Williamson, 2017). Would individuals approach the task of judging the morality of football players taking a knee during the national anthem the same way they would approach the task of judging football players’ athleticism during the game? Our findings suggest not. Even though we found the emotional reactions to such different forms of excellence to be equivalently positive, these reactions involved distinct cognitive, behavioral and neural processing. This work aligns with social psychological research describing two ways of perceiving others’ actions: by focusing on their abstract meaning or on their concrete, observable features (Liberman and Trope, 2008; Trope and Liberman, 2010). Further, the discovery of individual differences suggests that some may develop a disposition toward abstract construals of actions—akin to a form of moral expertise—that would be worth investigating further.

This study has some limitations. First, we did not pre-register our hypotheses (though our hypotheses were a priori) and the results are yet to be replicated by another laboratory. Second, gaze aversion and neural activation were not measured simultaneously. Our methodology is, however, unique in combining coding of natural behavior in an interview setting that closely resembles real-life storytelling interactions, with neuroscientific analysis. Neurobiological correlates of cognition and emotion are usually reported in the absence of behavioral data, and neural data are rarely connected to natural, real-world reactions to social stimuli (Immordino-Yang, 2010, 2013).

The feelings that arise from watching others’ acts of bravery, justice and self-sacrifice are among the most powerful motivators of human moral behavior (Haidt, 2003; Immordino-Yang and Sylvan, 2010; Schnall *et al*., 2010). The meaning of these virtuous actions transcends their immediate context to illustrate emulatable qualities of character that apply broadly and can be profoundly inspiring. Discerning these qualities appears to involve a neural mechanism that facilitates disengaging from the physical context. ‘Looking up’ (or away) may therefore support individuals in appreciating the intangible beliefs and values that comprise moral virtue.

## Funding

This work was supported by a Santander Cambridge Scholarship to G.P.; ESRC grant RES-000-22-4453 to S.S.; NSF CAREER grant 1151920, and support from the USC Provost, Brain and Creativity Institute and Rossier School of Education to MHI-Y.
